# The Rise of Valley Fever: Prevalence and Cost Burden of Coccidioidomycosis Infection in California

**DOI:** 10.3390/ijerph16071113

**Published:** 2019-03-28

**Authors:** Leslie Wilson, Jie Ting, Harold Lin, Rahil Shah, Michael MacLean, Michael W. Peterson, Nathan Stockamp, Robert Libke, Paul Brown

**Affiliations:** 1 Department of Clinical Pharmacy, University of California, San Francisco, CA 94118, USA; jie.tingjie@gmail.com (J.T.); rahil.shah@ucsf.edu (R.S.); 2 Kaiser Permanente Fresno, Fresno, CA 93720, USA; Harold.H.Lin@kp.org; 3 Kings County Department of Public Health, Hanford, CA 93230, USA; maclean.mlm@gmail.com; 4 Department of Medicine, University of California, San Francisco (UCSF), Fresno, CA 93701, USA; mpeterson@fresno.ucsf.edu (M.W.P.); nstockamp@fresno.ucsf.edu (N.S.); rlibke@fresno.ucsf.edu (R.L.); 5 Department of Social Sciences, Humanities and Arts, University of California, Merced, CA 95343, USA; pbrown3@ucmerced.edu

**Keywords:** coccidioidomycosis, California, cost-of-illness, economic analysis, Valley fever, infection, cost, prevalence

## Abstract

Coccidioidomycosis (CM) is a fungal infection endemic in the southwestern United States (US). In California, CM incidence increased more than 213% (from 6.0/100,000 (2014) to 18.8/100,000 (2017)) and continues to increase as rates in the first half of 2018 are double that of 2017 during the same period. This cost-of-illness study provides essential information to be used in health planning and funding as CM infections continue to surge. We used a “bottom-up” approach to determine lifetime costs of 2017 reported incident CM cases in California. We defined CM natural history and used a societal approach to determine direct and discounted indirect costs using literature, national datasets, and expert interviews. The total lifetime cost burden of CM cases reported in 2017 in California is just under $700 million US dollars, with $429 million in direct costs and $271 million in indirect costs. Per person direct costs were highest for disseminated disease ($1,023,730), while per person direct costs were lowest for uncomplicated CM pneumonia ($22,039). Cost burden varied by county. This is the first study to estimate total costs of CM, demonstrating its huge cost burden for California.

## 1. Introduction

Coccidioidomycosis (CM), also known as Valley fever (VF), is a fungal infection endemic in the southwestern portion of the United States (US; especially California and Arizona), as well as in parts of Central and South America [1,2]. CM infection is caused by inhalation of spores (arthroconidia) that become airborne from soil disturbances in endemic areas [2,3]. In California, reported CM incidence increased from 6.0/100,000 in 2014 to 18.8/100,000 in 2017, representing a >213% increase in the number of cases. More so, incidence rates in the first half of 2018 are almost double compared to the same time frame in 2017, showing a continual rise in infections [4]. The reasons for this are not well established. Given the precipitous rise in the number of infections, the lifetime treatment of CM will be an increasing cost burden to the state of California.

Our goal is to model CM and its complications and to estimate the average lifetime healthcare utilization and cost (direct and indirect) associated with CM among incident CM cases in California in 2017. The lifetime model of CM estimates that 60% of CM cases are asymptomatic, while the remaining 40% may experience a self-limiting respiratory illness, life-threatening pneumonia, meningeal infection, or dissemination [5]. The more severe disease manifestations often require long-term use of antifungal medications and incur significant healthcare utilization [6]. Costs include direct and indirect factors. Direct costs of CM include those for diagnosis, initial and follow-up physician visits, treatment procedures, hospitalizations, medication, and home or nursing home care. Indirect costs include days of work lost, morbidity disability payments, and mortality costs.

To our knowledge, only two economic studies were published on CM: a cost-effectiveness study of a potential CM vaccine, and total charges of CM-associated hospitalizations in California [7,8]. In our study, we model and estimate total lifetime costs of CM and its health consequences in California. Data from our study can help policy-makers understand the importance of CM, recognize areas of healthcare most affected by CM, and prioritize allocation of resources for treatment and prevention policies.

## 2. Materials and Methods

This cost-of-illness study used a “bottom-up” approach to estimate the lifetime costs of all 2017 reported incident cases in California. We firstly modeled the natural history of the disease, and then calculated lifetime costs by identifying specific items of healthcare use. The frequency of healthcare use was then multiplied by the proportion of cases utilizing the item of healthcare, and the costs of each item. We used a societal approach and determined both direct and indirect costs in 2017 US dollars and discounted lifetime indirect costs to present value. Our data were obtained from literature, national datasets, and expert interviews (Drs. Harold Lin, Michael MacLean, Michael Peterson, Nathan Stockamp, Robert Libke, personal communication, January–August 2014), and was exempted by the University of California, San Francisco (UCSF) Institutional Review Board. Expert interviews were used to better understand the natural progression of valley fever and helped guide treatment algorithms. A literature-based initial lifetime treatment model was modified by each expert based on their clinical experience (see Figure S1, Supplementary Materials). Experts each estimated population probabilities at each point in the model based on their extensive experience and knowledge treating coccidioidomycosis infections, especially in endemic areas of California. Any differences among experts were then discussed, and a consensus was reached on the best estimates before national costs were applied. These methods are used frequently, in cases such as these, to supplement estimates where literature and databases lack the necessary detailed information [9].

### 2.1. Study Population

Our study population was 2017 reported CM cases in California (*n* = 7466) (the latest data available). We estimated the expected lifetime cost of CM for our study population based on the natural history of CM and its published treatment guidelines (Table 1). To address the gap in literature on the healthcare utilizations of CM patients and expected loss in productivity from the disease, we conducted guided interviews with CM treatment experts from endemic settings in California. The guided interviews were one to two hours of discussion with each expert separately using a literature-based, itemized spreadsheet of disease epidemiology, natural history, diagnosis, treatment, and follow-up. Experts were asked questions regarding exact population probabilities at each point of the natural history disease model. Healthcare utilization of the pre-determined treatment options derived from literature review were also estimated based on their treatment experience and records.

To construct a natural history model for the disease, we firstly identified the major manifestations of CM from the literature and expert opinion: (1) uncomplicated pneumonia, (2) diffuse and/or chronic pneumonia with no dissemination, (3) disseminated disease, including meningitis, and (4) other changes in chest, including chronic pulmonary nodules, and chronic pulmonary cavity. We determined the proportion of patients experiencing each CM manifestation.

**Figure 1:**
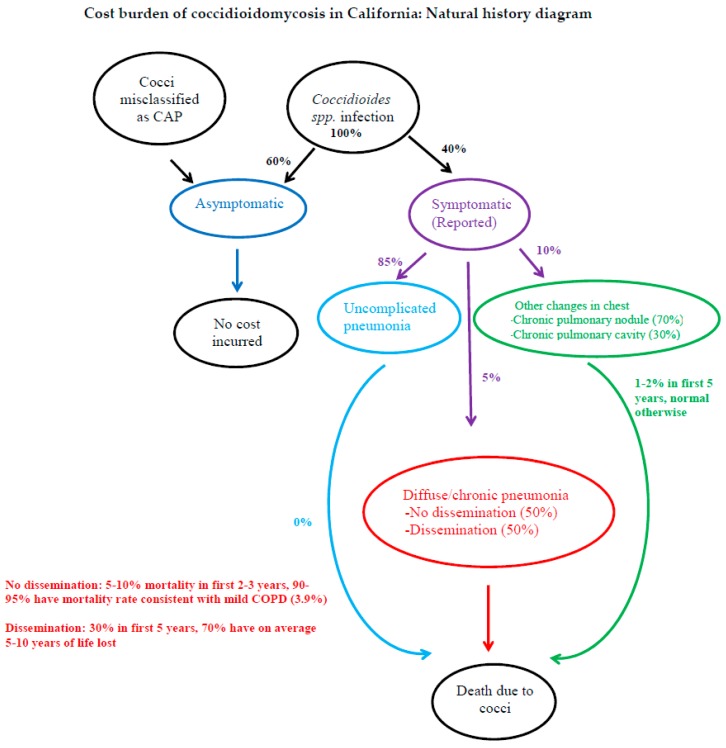
Coccidioidomycosis natural history. CAP = community-acquired pneumonia; spp = forma specialis.

Our natural history model (Figure 1) has several analytic assumptions. We assumed that 60% of annual CM cases are asymptomatic and, therefore, not reported. All asymptomatic cases were assumed to recover and develop immunity against CM and, therefore, did not seek healthcare nor incur any cost. Secondly, it is assumed that the annual reported cases represent the remaining 40% with symptoms of varying degrees, consistent with literature [5,6]. Finally, patients with CM misclassified as community-acquired pneumonia were included in the healthcare and diagnosis and treatment costs incurred before correct CM diagnosis [14,15,16].

This figure shows the natural history of Coccidioidomycosis based on published literature [2,4,5,14], treatment guidelines [12], and expert interviews (Harold Lin, Michael MacLean, Michael Peterson, Robert Libke, Nathan Stockamp, personal communication, January–August 2014).

Among the symptomatic cases, ~85–90% will have uncomplicated, self-limiting pneumonia, ~2.5% will have diffuse or chronic pneumonia without dissemination, and ~2.5% will have disseminated disease, including meningitis, while the remaining ~10% will have other chest changes (70% chronic pulmonary nodule, 30% chronic pulmonary cavity). Among those with diffuse/chronic pneumonia without dissemination, 5–10% will die in the first 2–3 years of having CM, while the remaining were assumed to have mortality rates consistent with mild chronic obstructive pulmonary disease. Among those with dissemination, including meningitis, 30% will die in the first five years of having CM, while the remaining will have, on average, 5–10 years of life lost. Among those with other changes in the chest, 1–2% will die in the first five years, while the remaining will have normal life expectancy. Lifetime burden of disease was calculated using an average disease onset at 46 years of age and an average life expectancy with a CM infection of 60 years [6].

### 2.2. Costs

We included both direct and indirect costs. Direct-care costs were those related to diagnosis, treatment, and follow-up of CM. Indirect costs were related to productivity losses due to CM. Categories of direct costs included diagnosis and treatment for respiratory symptoms before CM diagnosis (“pre-CM diagnosis”), CM diagnosis, initial and follow-up physician visits, treatments, procedures, hospitalizations, medications, and home or nursing home care. Data on CM-associated hospitalizations was obtained from the California patient discharge dataset 2017 using ICD-10 B.38.0-B.38.4, B38.89, and B38.9. Per person lifetime costs were the sum of lifetime direct and indirect costs. Total lifetime cost for California was calculated by multiplying the number of patients in each disease manifestation by their respective per person lifetime costs.

National costs sources included the Centers for Medicare and Medicaid Services CPT (Current Procedural Terminology) codes for costs of diagnosis, physician visits, and treatment procedures, the average wholesale price (AWP) of 17% for contract pricing using Red Book Online (Truven Health Analytics) for medication costs, and the Healthcare Cost and Utilization Project (HCUP) for national hospitalization costs by ICD-10 codes for CM [17,18]. Home-care costs were obtained from the US Bureau of Labor and Statistics (BLS), and nursing home costs were obtained from the US Department of Health and Human Services [19]. Indirect costs were salaries and compensation from the Bureau of Labor Statistics [20].

Indirect costs included work loss, short- and long-term disability, and mortality costs. The human capital approach was used to value lifetime productivity losses due to work loss and mortality. Estimates from Federal Reserve Economic Data were used to determine the average annual hours worked (1758), multiplied by average hourly compensation, wages, and salary ($32) for each day lost on an annual basis ($56,798 from 2012 to $59,214 in 2016) [21,22]. Compensation was increased annually to reflect projected increases to wages and salaries (1.6%) and to compensation (2.1%) until 2014. From 2015 to 2020, we increased compensation, wages, and salary by 1.55% per year, based on BLS projections. From 2020 to 2024, we estimated a 2% increase based on assumed easing of the economic downturn by then. An annual unemployment rate from 2012 to 2016 (10.4 to 8.0%), 8% until 2019, and 7% after 2020 was used to decrease the proportion with productivity losses, and as the economy is expected to improve slowly over time. Disability payments included the average short-term disability payments for California in 2016, an average of $488/week for 16 weeks [23]. Long-term disability payments included Supplemental Security Income (SSI) for those with disseminated disease. We assumed long-term disability was received for 10 years, and discounted these payments by the current discount rate (1%) [24]. Average SSI payments included both Federal and State payments for 2016 ($552/month) and, beyond that, they were increased annually by 1.4%.

Final cost burden was the total of all direct and indirect lifetime costs per reported CM case and across all reported cases in California. The base case indirect cost estimates were inflated for increases in healthcare costs and discounted to present dollars. Direct costs were not discounted because most costs occur in the first two years. Direct costs beyond the first year (e.g., hospitalizations) were also not discounted as these were estimated to occur during a person’s lifetime, but were not attributable to an exact future year.

Multiple one-way sensitivity analyses were also conducted. Ranges of ±10% were used to vary the percentage of individuals that were shown to be symptomatic and, thus, an incident case, as these cases may be under- or over-reported. Also, the number of individuals in each disease manifestation was adjusted to reflect any potential errors in reporting or misdiagnosis. Lastly, medication costs were identified as a major source of cost burden, especially for those who had disseminated disease and, therefore, were subject to sensitivity analysis by varying their costs by ±25% of the base case.

## 3. Results

The estimated total lifetime cost burden of all the CM cases reported in 2017 (*n* = 7466) in California is just under $700 million (discounted), with $429 million in direct costs and $271 million in indirect costs (Table 2).

### 3.1. Direct Costs

The average lifetime direct costs per person across all manifestations of CM are high ($57,413). Accounting for the frequency of each manifestation of the disease, CM with dissemination, including meningitis, still accounts for the highest total direct costs (over $191 million), followed by those with CM-associated uncomplicated pneumonia (85% of reported cases), which cost almost $140 million. The other CM manifestations contributed less to the overall total California costs.

#### 3.1.1. Uncomplicated Pneumonia

CM-associated uncomplicated pneumonia incurred the lowest per person lifetime direct costs ($22,039) among all CM disease manifestations (Table 2). This included a pre-CM diagnosis period with an estimated three physician visits and a course of antibiotics. We also assumed 50% of these pre-CM diagnosis cases had a second course of antibiotics, and that 23% firstly sought healthcare in the emergency department (ED) [10]. The initial diagnosis of CM consisted of an immunodiffusion (ID) serologic test and complement fixation (CF) titer, plus a chest X-ray for all patients with uncomplicated pneumonia. An estimated 25% of patients would require a second ID and CF titer, as well as a chest computerized tomography (CT). The largest areas of cost for CM-associated uncomplicated pneumonia were hospitalizations ($13,027) and medications ($6891). We estimated 70% would not require hospitalization, while the remaining 30% would require hospitalization at least once, with fewer patients needing more than one hospitalization due to CM in their lifetime. Fluconazole is the preferred treatment for all CM cases, but the dose and length of treatment varied by type of disease manifestation. Other antifungals used included itraconazole, amphotericin B (primarily in pregnancy or severe CM cases), and voriconazole, if failing fluconazole treatment. In uncomplicated pneumonia, antifungal treatment lasted six months and lifetime medication costs were $6891 per person. We assumed a regime for follow-up costs which primarily included repeat ID testing and titers and chest X-rays, which varied by disease group.

#### 3.1.2. Diffuse/Chronic Pneumonia

CM-associated diffuse/chronic pneumonia without dissemination is much more costly per person than uncomplicated pneumonia, primarily due to increased risks of hospitalization over their lifetime, and longer use of antifungals (average, 36 months). Follow-up testing is also more intense with CM-associated chronic/diffuse pneumonia. Physicians interviewed suggested they ideally follow the disease progression with ID and titers and chest X-rays at three-month intervals for the first 12 months, but that (many) patients do not fully comply with this recommendation. Therefore, we also accounted for the expected loss to follow-up in our costing (Table 2). Total lifetime per person direct costs for CM-associated diffuse/chronic pneumonia without dissemination are about $132,000, five times higher than for CM-associated uncomplicated pneumonia.

#### 3.1.3. Disseminated Disease, Including Meningitis

CM infections can sometimes lead to severe disseminated disease (~1% of total CM infections) [5]. Lifetime direct costs per person of CM-associated dissemination, including meningitis, were $1,023,730, almost eight times as high as for CM-associated pneumonia without dissemination. Here, diagnosis confers a greater morbidity and is more costly as more organs can be affected, including the skin, bones, joints, and the central nervous system (CNS). Estimated lifetime hospitalization costs were especially high for these patients ($672,730). Additionally, those with disseminated disease require lifelong antifungals, totaling $321,899 per person.

Also contributing to the high costs of CM-associated dissemination, including meningitis, is the use and revision of ventriculoperitoneal shunts. Patients with CNS infections or coccidioidal meningitis are at risk of complications including hydrocephalus. Shunts are used therapeutically to relieve hydrocephalus that occurs in approximately 30–50% of coccidioidal meningitis [25]. In some cases, shunts may be used for administration of medication. These types of shunts commonly fail, and many patients require one or more surgical revisions [26].

Finally, additional costs were accrued due to frequent follow-up testing, as well as requirements for temporary home care and short- and long-term nursing home placement for a small proportion of individuals with disseminated disease. This results in average lifetime per person costs of over $1 million ($1,023,730), quite a significant burden for an individual.

#### 3.1.4. Pulmonary Nodules or Cavities

The estimated direct costs for treating individuals with CM-associated pulmonary nodules or cavities were surprisingly high. These patients are sometimes diagnosed with CM only when a nodule is found on a chest X-ray done for another medical reason. This results in the need to conduct a diagnostic lung cancer work-up to rule out lung cancer in 90% of these individuals, as indicated by expert opinions. This work-up is costly (~$76,000), and may be an area for cost savings if characteristics of the nodule or cavity can be better targeted to CM rather than cancer. The total estimated lifetime per person direct costs for CM with pulmonary nodule are $95,399, and they are slightly higher with a pulmonary cavity ($101,748) because of additional bleeding complications from cavity ruptures.

### 3.2. Indirect Costs

Estimated total indirect costs included work loss, short-term and long-term disability payments, and productivity losses due to mortality (Table 3).

#### 3.2.1. Early Work Loss

We estimated an average total work loss of seven days for CM-associated uncomplicated pneumonia, and for CM-associated pulmonary nodules and cavities, while an estimate of 43 days of work loss was obtained among CM-associated diffuse/chronic pneumonia patients without dissemination. Among CM-associated dissemination, we estimated 10% had permanent work loss, and the remaining 90% had an average work loss of 90 days. Work-loss costs were lowest for those with uncomplicated pneumonia, and those with nodules or cavity ($931), and highest for those with diffuse/chronic pneumonia and those with dissemination ($11,971–$15,961).

#### 3.2.2. Disability

Those with diffuse/chronic pneumonia had an average estimated 16 weeks of short-term California disability payments, and 10% of those with dissemination had 10 years of long-term disability payments. This resulted in total disability payments across all CM patients of $4,156,447 (discounted), of which more than half were for those few patients with disseminated disease ($2,697,101).

#### 3.2.3. Mortality

Mortality and life expectancy were estimated by disease manifestation group. We assumed a normal life expectancy for those with CM-associated uncomplicated pneumonia, resulting in no productivity losses due to mortality. Among those with CM-associated diffuse/chronic pneumonia without dissemination, mortality was estimated to be 5–10% per year for the first three years, while the remainder (90–95%) had a mortality rate consistent with mild chronic obstructive pulmonary disease (COPD) (35% in 10 years, ~4.3% per year) [27]. We estimated the mortality of those with disseminated disease at 30% per year for the first five years and then had twice the mortality rate of mild COPD (~8.6% per year) for the next five years. Again, mortality wage loss costs were estimated to account for 10 years of additional life expectancy lost compared to normal life expectancy. These lost productivity wages were increased for wage inflation and discounted for net present value estimates. Mortality for those with pulmonary nodules and cavities was 1% per year in the first five years with 10 years of lost productivity for those dying. Those not dying in the first five years were assumed to have a normal life expectancy.

Estimated total lifetime mortality costs were highest for those with disseminated CM ($531,907). Those with nodules or cavities, diffuse/chronic pneumonia without dissemination, and those with dissemination, including meningitis, had mortality costs ranging from $125,952–$531,907.

#### 3.2.4. Costs by County

We estimated cost burden by county by calculating total CM costs in endemic counties (Fresno, Kern, Kings, Madera, San Luis Obispo, and Tulare), as well as those with >100 reported CM cases in 2017 (Table 4) [4,8]. Cost burden ranged from $6.0–$13.3 million for the two counties with the lowest cost, to $87.5–$257.5 million for the two counties with the highest cost burden. Three other counties, although not classified as endemic, also had a high cost burden: Los Angeles ($87.5 million), San Diego ($13.3 million), and Monterey county ($17.1 million). Madera, although endemic, had the lowest costs of this group ($6.1 million).

In sensitivity analyses, the percentage of individuals that were classified under each disease manifestation was varied by a 10% range above and below the total number of individuals that were reported to have CM in California in 2017. From an estimated base of 60% of CM cases being asymptomatic, and again varying by 10% above and below this rate, the total direct and indirect lifetime costs to California can span from $874 million to $524 million. We also adjusted by 10% the percentage of CM cases presenting as uncomplicated pneumonia. This variation from 76.5–93.5% gave a range of total lifetime costs to California from $314 million to $1.1 billion.

Medication costs in our base case estimates were also a significant source of cost burden in the treatment of CM. Varying lifelong medication costs for those who have disseminated disease ±25% changed the total lifetime direct and indirect costs of CM in California from $720 million to $690 million.

## 4. Discussion

The estimated total cost burden of CM in California is high, with about $429 million in direct and $271 million in indirect costs for the lifetime of cases reported each year. Direct costs are higher than the indirect costs, accounting for about 61% of total costs. The large indirect cost burden rests primarily on a small number of cases with disseminated disease, although those with chronic/diffuse pneumonia without dissemination also share in this indirect cost burden.

Lifetime per person costs of CM in California were $94,000, with $58,000 direct and $36,000 indirect costs. With the recent annual increase in number of new cases in California, the total costs likely will increase over time. For example, reported incidence from January through July 2018 showed an increase of almost 75% compared to the same months in 2017, although reported cases are lower again in August of 2018 [4]. The CM cost burden is not shared equally among California counties. Although the defined endemic counties generally experience the highest cost burden, this is not always the case. For example, Madera County (defined as endemic) has a small population and small cost burden, while three non-endemic counties have relatively high cost burdens. Patients with CM who are uninsured will need to rely on county or hospital funds for their care. The county-specific CM cost burden should be considered when estimating healthcare budgets in these counties.

The economic burden of CM is greater than many other region-specific diseases. For example, estimated lifetime direct per person costs of Chagas disease (a parasitic disease found in southern regions of United States and Latin America) was only $29,111, about half the costs of CM [28]. Lyme disease has a direct annual per person cost of $3953 (2017 dollars), with an estimated total annual cost of $3.2 billion [29]. The average per person costs of treating multidrug-resistant TB (MDR-TB) is $140,000, increasing to $452,000 for extensively drug-resistant TB, only about half of the direct costs of CM treatment [30]. However, adding productivity losses to treatment costs for MDR-TB brings the estimated cost per case to $583,000, which is much higher than the $94,000 per person total direct and indirect costs of CM. Lifetime total costs of care per human immunodeficiency virus (HIV)-infected patient ($400,000) is also higher than for CM, although costs per breast cancer patient ($21,000–$105,000) is more similar to CM [31,32]. Although differing methodologies to calculate costs were used in these studies, these comparisons in understanding the overall cost burden of CM remain relevant.

Average CM total lifetime costs and per person lifetime costs are high ($700 million and $94,000, respectively). Disseminated cases, including meningitis, incur the highest cost due to long-term treatment needs. Cancer work-up for nodules and cavities offers an opportunity for cost control. Decreasing the cost of the 90% of patients receiving a complete cancer work-up by half could save almost $38,000 per person in this CM group ($196,000 to $120,000). This could also reduce the total California lifetime CM costs by $28 million ($700–$672 million). The direct cost of treatment is 61% of total costs, likely due to the high cost of repeat hospitalizations and antifungal treatment over many years. Indirect costs are also high and may be underestimated given the lack of strong mortality and life expectancy data for CM cases. The availability of more accurate data on mortality would improve these estimates. The disseminated cases, although rare, account for much of the direct and indirect costs, given their long treatment needs and higher mortality and disability. Uncomplicated pneumonia cases account for a high proportion of the direct costs as well, due to their high volume compared to the other groups.

It is important to note that our experts expressed that CM cases are a diverse group of patients, with a large variation in utilization and costs by geographic region, as well as in their availability of insurance and, therefore, access to care. They describe that there are many cases of CM in undocumented workers who are not currently insured, and who are receiving care from state-run clinics or accessing free care. They also indicated that these workers are less likely to seek care or follow-up on their recommended treatment over time. It is important to examine some of these variations in population type and subsequent care in future studies.

Our study has a few limitations. Firstly, we assumed that all symptomatic cases and, therefore, those who incurred costs were reported. While symptomatic cases could remain misclassified as community-acquired pneumonia, some reported cases may also be asymptomatic or are false positives [14,15,16]. More accurate estimation on the number of symptomatic cases among the reported cases is needed in California. Secondly, we estimated that 2.5% of our population have dissemination, including meningitis, consistent with literature [5,33]. However, a recent study reported that up to 8% of CM infections could progress to disseminated disease [34]. This would substantially increase the total lifetime cost of disseminated disease. More accurate estimation of the risk of dissemination is, therefore, needed. Thirdly, our experts expressed that there was considerable variation in the use of shunts. We estimated that 15% of those with meningitis would require shunt placement to relieve hydrocephalus. Although hydrocephalus occurs in 30–50% of coccidioidal meningitis, we are unsure how many would require or receive a shunt [25]. Although the estimated lifetime cost per person of shunt placement/replacement in our population is low (<$8000), the cost of shunt placement to a patient requiring the procedure, including hospitalization, is high (~$52,000). It is, therefore, important to be able to more accurately estimate the frequency and value of shunt placement. Additionally, as information regarding medical services provided was partly obtained through expert interviews, this method may skew cost calculation based on a practitioner’s opinion. However, expert panel interviews were shown to be useful in previous cost-of-illness studies for diseases with undefined treatment pathways when this is the best available evidence source, especially for outpatient expenses [35,36]. As our study is the first to calculate the total lifetime cost of CM in California, it may be used as a stepping stone for additional studies which should incorporate the use of medical claims data. Claims data are limited for calculation of lifetime costs for this disease, however, since many cases present only as uncomplicated pneumonia. Finally, there is some debate about the helpfulness of cost-of-illness studies to bring about policy change toward a specific disease. Other economic methods such as cost-effectiveness analyses can also be helpful, but focus more on comparing the economic value of different treatment approaches. Cost-of-illness studies can provide a detailed overview of the factors that contribute toward a disease and its progression, as well as the associated costs. Our cost-of-illness study can inform later cost-effectiveness studies and can allow for potential interventions to be tested and prioritized given the disease specific data obtained from the research. Cost-of-illness studies may also provide a better estimation of population health and allow for insight into potential health gaps for a certain disease [37,38].

Future studies can examine costs in more depth, using utilization and cost data from larger databases in California, to provide more assurance of the accuracy of our cost estimates. Future studies should also include the rapid diagnostic test (by DxNA) approved in December 2017 with the potential to shorten testing times, and allow preventative testing of those going into high risk areas. If rapid testing for antibodies prior to starting work in high risk areas was the standard, appropriate risk mitigation strategies (NIOSH-certified breathing mask or relocation) could be taken to reduce the chance of a CM infection in those at highest risk [39,40,41].

## 5. Conclusions

The National Institute of Health, Institute of Medicine, and the US Congress recognize the importance of cost-of-illness studies in setting research priorities [42]. This cost-of-illness study can help define the magnitude of the disease in dollars, and provide an important guide and resource justification for policy development, priority setting, and management of public health. As coccidioidomycosis infections continued to rise in California in the first half of 2018, our detailed analysis defining its high economic impact gains enhanced relevance. This study models the natural history of Valley fever, detailing the specific areas of highest cost burden, and providing cost estimates for the first time. Future economic research will need to address the high potential to reduce overall healthcare expenditure, with adoption of the newly available diagnostic measures and enhanced mitigation strategies for those at most risk in endemic areas of California.

## Figures and Tables

**Table 1:** Estimated direct lifetime costs per person, stratified by types of costs and coccidioidomycosis (CM) disease manifestation in California.

Cost Type	Item	Utilization	Average Per Person Lifetime Cost ^1^	Reference
**Uncomplicated pneumonia**				
*Pre-CM diagnosis* ^2^				
Physician visit		100% had 3 physician visits	$487	[10] ^3^
ER visit		23% first sought care in ER	$17	
Medication	Azithromycin/levaquin	100% (50% will require 2nd course)	$114	
*Diagnosis*	Immunodiffusion and titer	100% (25% will require repeat testing)	$322	[11] ^3^
	Chest X-ray	100%		
	Chest CT	25%		
	Others, HIV testing	100%		
*Post-CM diagnosis*				
Hospitalization	Requiring hospitalization	40%	$13,027	^3^
	1 lifetime hospitalization	90%		
	2 lifetime hospitalizations	7%		
	>2 lifetime hospitalizations	3%		
Medication	Fluconazole (400 mg/day)	90% (6 months)	$6891	[12] ^3^
	Itraconazole (200 mg twice/day)	3% (6 months)		
	Amphotericin B (3 mg/kg/day)	2% (for pregnant women, 6 months)		
	Voriconazole (200mg twice/day)	5% (after failing fluconazole/voriconazole, 6 months)		
Follow-up	Immunodiffusion and titer	100% every 3 months for 12 months (expected compliance 50–80%)	$1181	^3^
	Chest x-ray			
Home care/nursing home		None	$0	Assumption ^3^
**Total**			**$22,039**	
**Diffuse/chronic pneumonia without dissemination**				
*Pre-CM diagnosis*		Same as for CM-associated uncomplicated pneumonia	$618	[10] ^3^
Other medication	4-drug regimen for tuberculosis	10% (1 month)	$17	
*Diagnosis*	Immunodiffusion and titer	100% (25% will require repeat testing)	$321	[11] ^3^
	Chest X-ray	50% have 2 X-rays/year outside of hospital		
	Chest CT	30% have chest CT outside of hospital		
*Post-CM diagnosis*				
Hospitalization	1st hospitalization in year 1	75%	$68,532	^3^
	2nd hospitalization in year 1	65% (of those with 1st hospitalization)		
	Hospitalization in year 2	100%		
Medication	Fluconazole (400 mg/day)	75% (36 months)	$55,313	[12] ^3^
	Itraconazole (200 mg twice/day)	25% (36 months)		
Follow-up	Immunodiffusion and titer	100% every 3 months for 12 months (expected compliance 50–80%)	$1566	^3^
	Chest X-ray			
	Chest CT	100% (at discharge, expected compliance 50–80%)		
Home care		100% (3 days a week for 3 months)	$2450	Assumption ^3^
Rehabilitation facility		100% (30 days)	$3599	
**Total**			**$132,416**	
**Dissemination, including meningitis**				
*Pre-CM diagnosis*		Same as for CM-associated uncomplicated pneumonia. 40% first sought care in ER	$618	[10] ^3^
*Diagnosis*				
Immunodiffusion/titer/chest X-ray/chest CT		Same as for CM-associated diffuse/chronic pneumonia without dissemination	$737	[11] ^3^
Lumbar puncture		50%		
MRI		15–20%		
Aspirates of joint effusions		10%		
Skin biopsy		10%		
Bone marrow biopsy		5%		
Lung biopsy		20%		
Lymph node biopsy		20%		
Liver biopsy		5%		
*Post-CM diagnosis*				
Hospitalization	1st hospitalization in year 1	100%	$672,730	^3^
	2nd hospitalization in year 1	65% (of those with 1st hospitalization)		
	Hospitalization after year 1	100% hospitalized once a year for life		
Medication	Fluconazole (1,000 mg/day)	98% (lifelong)	$321,899	[12] ^3^
	Amphotericin B (3 mg/kg/day)	2% (lifelong)		
Other treatment considerations	Ventriculoperitoneal shunt placement	15% of those with meningitis	$7691	[12] ^3^
	Ventriculoperitoneal shunt replacement	100% of shunts replaced once in lifetime		
Follow-up	Immunodiffusion and titer	100% (every 3 months in year 1, every 6 months for life; MRI every 6 months for life; lumbar puncture 2 times in year 1, 5 times in lifetime; expected compliance 50–80%)	$16,877	^3^
	Chest X-ray			
	Chest CT			
	Liver function test			
	Renal function test			
	MRI			
	Lumbar puncture			
Home care		100% (3 days a week for 3 months)	$2450	Assumption ^3^
Nursing home	Temporary stay	10% (2 months)	$728	
**Total**			**$1,023,730**	
**Other changes in chest, pulmonary nodule**				
*Pre-CM diagnosis*		Same as for CM-associated uncomplicated pneumonia	$618	[10] ^3^
*Diagnosis*	Immunodiffusion and titer	100% (25% will require repeat testing)	$76,631	[11,13] ^3^
	Chest X-ray	100%		
	Chest CT	25%		
	Diagnostic work-up for lung cancer *(CT scan, biopsy if indeterminate, watch and wait if benign)*	90%		
*Post-CM diagnosis*				
Hospitalization		Same as for CM-associated uncomplicated pneumonia	$13,027	^3^
Medication	Requiring medication	25%	$1561	[12] ^3^
	Fluconazole (400 mg/day)	90% (6 months)		
	Itraconazole (200 mg twice/day)	5% (6 months)		
	Voriconazole (200 mg twice/day)	5% (after failing fluconazole/voriconazole, 6 months)		
Follow-up	Immunodiffusion and titer	100% every 3 months for 12 months, then every 6 months for 1 year (expected compliance 50–80%)	$3562	^3^
	Chest X-ray			
Home care/nursing home		None	$0	Assumption ^3^
**Total**			**$95,399**	
**Other changes in chest, pulmonary cavity**				
*Pre-CM diagnosis*		Same as for CM-associated changes pulmonary nodule	$95,399	[10,11] ^3^
*Diagnosis*				
*Post-CM diagnosis*				
*Additional hospitalization*				
Cavity complications		5%	$4207	^3^
Hemoptysis/chest pain		5–10%	$2142	^3^
Home care/nursing home		None	$0	Assumption ^3^
**Total**			**$101,748**	

CT = computerized tomography; HIV = human immunodeficiency virus; MRI = magnetic resonance imaging; ER = emergency room. ^1^ Drug costs were from Red Book Online (Truven Health Analytics); medical procedure costs from Centers for Medicare and Medicaid Services CPT (Current Procedural Terminology) codes. ^2^ Applies to all CM disease manifestations. ^3^ Harold Lin, Michael MacLean, Michael Peterson, Robert Libke, Nathan Stockamp, personal communication.

**Table 2:** Estimated total direct and indirect lifetime costs, stratified by types of costs and coccidioidomycosis (CM) disease manifestation, for incident CM cases in 2017 (*n* = 7466) in California.

CM Disease Manifestation	Number (*n* = 7466)	Average Per Person Lifetime Cost	Total Lifetime Cost for California
**Direct costs**			
Uncomplicated pneumonia	6346	$22,039	$139,859,494
Diffuse/chronic pneumonia without dissemination	187	$132,416	$24,761,792
Dissemination, including meningitis	187	$1,023,730	$191,437,510
Other changes in chest, pulmonary nodules	522	$95,399	$49,798,278
Other changes in chest, pulmonary cavity	224	$101,748	$22,791,552
**Indirect costs**			
Uncomplicated pneumonia	6346	$931	$5,908,126
Diffuse/chronic pneumonia without dissemination	187	$350,063	$65,461,781
Dissemination, including meningitis	187	$562,291	$105,148,417
Other changes in chest, pulmonary nodules	522	$126,883	$66,232,926
Other changes in chest, pulmonary cavity	224	$126,883	$28,421,792
**Total costs of CM**			
Direct costs			$428,648,626
Indirect costs			$271,173,042
Work loss			$11,825,936
Disability			$4,156,449
Mortality			$255,190,657
Total direct + indirect costs			$699,821,668

**Table 3:** Estimated indirect lifetime costs per person, stratified by types of costs and coccidioidomycosis (CM) disease manifestation in California.

Cost Type	Frequency and Utilization	Average Per Person Lifetime Cost ^1^	Reference
Uncomplicated pneumonia			
Work loss	7 days	$931	[10] ^5^
Disability	None	$0	^5^
Mortality	Normal life expectancy	$0	
Total		$931	
Diffuse/chronic pneumonia without dissemination			
Work loss	90 days	$11,971	[10] ^5^
Disability	16 weeks short-term CA disability	$7804	^5^
Mortality ^2^	5–10% death in first 2–3 years	$330,288 ^3^	
	Remainder have mortality rates consistent with mild COPD (4.3% annually)		
Total		$350,063	
Dissemination, including meningitis			
Work loss	120 days	$15,961	[10] ^5^
Disability	16 weeks short-term CA disability	$7804	^5^
	Long-term social security payment	$6619 ^3,4^	
	State payments for those with permanent disability		
Mortality ^2^	30% death in first 5 years.	$531,907 ^3^	^5^
	Remainder have mortality rates consistent with twice that of mild COPD (8.6% annually)		[27] ^5^
Total		$562,291	
Other changes in chest, pulmonary nodule			
Work loss	7 days	$931	^5^
Disability	None	$0	
Mortality ^2^	1% death in first 5 years. Remainder have normal life expectancy	$125,952	
Total		$126,883	
Other changes in chest, pulmonary cavity			
Work loss	7 days	$931	^5^
Disability	None	$0	
Mortality	1% death in first 5 years. Remainder have normal life expectancy	$125,952	
Total		$126,883	

COPD = chronic obstructive pulmonary disease; CA = California. ^1^ Drug costs were from Red Book Online (Truven Health Analytics); medical procedures costs from Centers for Medicare and Medicaid Services CPT codes. ^2^ We assumed all deaths have an average 10 years of life lost. ^3^ Discounted at 1% per year. ^4^ Average payment increase of 1.4% per year. ^5^ Harold Lin, Michael MacLean, Michael Peterson, Robert Libke, Nathan Stockamp, personal communication.

**Table 4:** Estimated total direct and indirect lifetime costs, stratified by endemic counties and counties with >100 reported coccidioidomycosis (CM) cases in 2017 in California.

County	Number (*n* = 7466)	Direct Cost	Indirect Cost	Total Cost
Fresno *	824	$47,308,662	$29,928,554	$77,237,216
Kern *	2748	$157,772,090	$99,810,276	$257,582,366
Kings *	260	$14,927,490	$9,443,476	$24,370,966
Los Angeles County	934	$53,624,138	$33,923,871	$87,548,009
Madera *	65	$3,731,873	$2,360,869	$6,092,742
Monterey	182	$10,449,243	$6,610,433	$17,059,676
San Diego	142	$8,152,706	$5,157,591	$13,310,297
San Luis Obispo *	419	$24,056,225	$15,218,525	$39,274,749
Tulare *	275	$15,778,692	$9,988,292	$25,776,983

* Coccidioidomycosis endemic counties.

## Supplementary Materials

The following are available online at https://www.mdpi.com/1660-4601/16/7/1113/s1: Figure S1: Physician interview guide and treatment model.
